# Multi-centennial fluctuations of radionuclide production rates are modulated by the Earth’s magnetic field

**DOI:** 10.1038/s41598-018-28115-4

**Published:** 2018-06-29

**Authors:** F. J. Pavón-Carrasco, M. Gómez-Paccard, S. A. Campuzano, J. F. González-Rouco, M. L. Osete

**Affiliations:** 1 Universidad Complutense de Madrid, 28040 Madrid, Spain; 2 Instituto de Geociencias IGEO (UCM-CSIC), 28040 Madrid, Spain; 3 Present Address: Istituto Nazionale di Geofisica e Vulcanologia (INGV), 00143 Rome, Italy

## Abstract

The production of cosmogenic isotopes offers a unique way to reconstruct solar activity during the Holocene. It is influenced by both the solar and Earth magnetic fields and thus their combined effect needs to be disentangled to infer past solar irradiance. Nowadays, it is assumed that the long-term variations of cosmogenic production are modulated by the geomagnetic field and that the solar field dominates over shorter wavelengths. In this process, the effects of the non-dipolar terms of the geomagnetic field are considered negligible. Here we analyse these assumptions and demonstrate that, for a constant solar modulation potential, the geomagnetic field exerts a strong modulation of multi-centennial to millennial wavelengths (periods of 800 and 2200 yr). Moreover, we demonstrate that the non-dipole terms derived from the harmonic degree 3 and above produce maximum differences of 7% in the global average radiocarbon production rate. The results are supported by the identification, for the first time, of a robust coherence between the production rates independently estimated from geomagnetic reconstructions and that inferred from natural archives. This implies the need to review past solar forcing reconstructions, with important implications both for the assessment of solar-climate relationships as well as for the present and future generation of paleoclimate models.

## Introduction

Nowadays, unravelling the external forcing changes that contributed to drive Holocene climate on the sub-millennial timescales is a major challenge. Solar irradiance changes are one of the important drivers of natural climate variability^1^ during this period. In this context, the quantification of the different mechanisms causing the variability of the production of cosmogenic radionuclides in the Earth’s upper atmosphere is crucial since Quaternary records of atmospheric cosmogenic nuclide production (e.g., ^10^Be or ^14^C) provide the only way to reconstruct past solar variability for periods preceding direct observations, i.e. the last 400 years^2^.

Cosmogenic radionuclides are produced in the upper troposphere and lower stratosphere (∼10–20 km altitude) by the cascade of nuclear reactions induced by high-energy charged particles (the so-called cosmic rays) that impinge on the Earth from all directions of space. Since cosmic rays are mainly composed of charged particles, a large fraction of them are deflected by the solar and the Earth’s magnetic fields. Consequently, the radionuclide production in the atmosphere is modulated by changes in the strength of the magnetic field of both the Sun and Earth. Increased magnetic fields cause a stronger magnetic deflection of cosmic rays and lower radionuclide production rates in the atmosphere and vice versa. However, the atmospheric concentration of cosmogenic isotopes also depends not only on strength of the geomagnetic field but also on its configuration, that greatly varies with time. These variations and their uncertainties need to be carefully considered for reconstructing past solar activity. Since variability in solar irradiance is related to changes in the solar magnetic field, solar forcing reconstructions can be derived from radionuclide production rates records corrected by the geomagnetic field effect. At this point, having reliable estimates of geomagnetic field changes and an effective means of correcting for their influence on isotope production become important.

The geomagnetic field influences radionuclide production rates by deflecting a part of the incoming cosmic rays, depending on their magnetic rigidity and incidence angle. These parameters along with the local magnetic latitude define a critical threshold, named cut-off rigidity, below which cosmic rays particles cannot penetrate into the Earth’s atmosphere. This leads to a latitudinal dependence of the cosmic ray fluxes and consequently of the production rate of cosmogenic nuclides. Thus, the higher the magnetic latitudes are, the larger the resulting production rates^3,4^. As mentioned, this mechanism is also controlled by the strength of the geomagnetic field, which can be represented by the dipole moment^5^ at global scale. The higher the dipole moment is, the stronger the deflection of the cosmic ray particles entering and, as a consequence, the lower the resulting radionuclide production^3,4,6^. The interaction between cosmic rays and the geomagnetic field has been analysed since 1950’s by direct measurements from ship and airborne studies. Results from these studies indicated that the cut-off rigidities are not consistent with an axial dipolar geomagnetic field and that contributions up to harmonic degree 8 should be considered to obtain a better agreement with cosmic ray observations^7,8^.

The above findings cannot be directly extrapolated to the past millennia since the interaction between cosmic rays, radionuclides and the geomagnetic field can only be studied from indirect measurements. The past radionuclide production can be recovered from terrestrial archives of isotope records (polar ice, trees and sediments^9^). The past evolution of the geomagnetic field before direct observations can be recovered from the magnetic signal recorded in volcanic rocks, archeological materials and sediments. A priori it should be therefore relatively straightforward to determine the solar activity during the Holocene, provided that the past radionuclide productions are known and that the intensity and configuration of the geomagnetic field in the past are well constrained^6^. However, in practice, it remains problematic because the determination of both processes is a long-standing issue that is challenging the scientific community nowadays^6,10–13^. As a consequence, estimates of past variations of solar variability during the Holocene are subject to considerable uncertainty^1^.

During the last decade, several works have investigated the impact of past geomagnetic field variations on radionuclide production with especial focus on the radiocarbon production rate^6,10–13^ (RcPR). These works used three types of paleomagnetic reconstructions. The first one assumes that local geomagnetic intensity records represent the true dipole moment at global scale^10^. The second, and most frequently used approach, obtains dipole moment reconstructions by means of regional weighted averages of paleointensity data^6,14,15^. This method, while offering an improved way to calculate the fluctuation of the Earth’s dipole moment, can introduce important biases through the averaging process due to the inhomogeneous temporal and spatial distribution of paleomagnetic data^15,16^. This averaging approach has been surpassed by geomagnetic spherical harmonic reconstructions^17–19^, which provide the most realistic approach to separate the geomagnetic field into the different harmonic contributions starting from the dipole field (first harmonic degree), the quadrupolar field (second degree) and subsequent harmonics. In this case, the dipole moment is estimated by the first harmonic degree and the magnetic latitudes can be derived from the inclinations at any point over the Earth’s surface.

The three methods mentioned before have been used to analyse the geomagnetic modulation of radionuclide production rates^6^. Previous studies suggest that the long-term variation (millennial time scale) of the global average RcPR is modulated by the geomagnetic field, with the solar magnetic field being the dominant actor of shorter wavelengths (centennial time scale). However, the geomagnetic field reconstructions considered present important smoothing effects due to the use of weighted averages or to the use of sedimentary paleomagnetic records for global geomagnetic field modelling purposes^19^. Sedimentary archives are generally used to improve the spatial and temporal coverage of paleomagnetic data during the Holocene, but they can only provide a smooth record of geomagnetic variations in comparison to volcanic or archaeological records^20,21^. Consequently, up to now, the geomagnetic field corrections used for reconstructing solar irradiance are prone to underestimate solar variability at multi-centennial to millennial timescales. This may have non negligible implications since there is a high-risk that important features in cosmogenic radionuclides records are being incorrectly attributed to non-geomagnetic effects with potential large impacts on our current understanding of past solar variability and, hence, in the external forcing factors used by the past^22,23^ and future^24^ generation of paleoclimate models covering the last millennia. In addition, the cited works^6,10,11^ assumed the simplest configuration of the geomagnetic field (i.e., they consider that it corresponds to a dipole field or an axial dipole field) to estimate the global average RcPRs, and a complete study about the effect of higher harmonic contributions remains to be done.

In this work, we propose a revaluation of the link between past geomagnetic field changes and the global average RcPR during the Holocene. To achieve this goal, we first reassess the influence of the geomagnetic harmonic degree in past global average RcPRs. Second, we present a complete statistical analysis of the covariability according to timescales and frequencies of global radionuclide production estimated from geomagnetic field models and independently from natural radionuclides archives.

**Figure 1:**
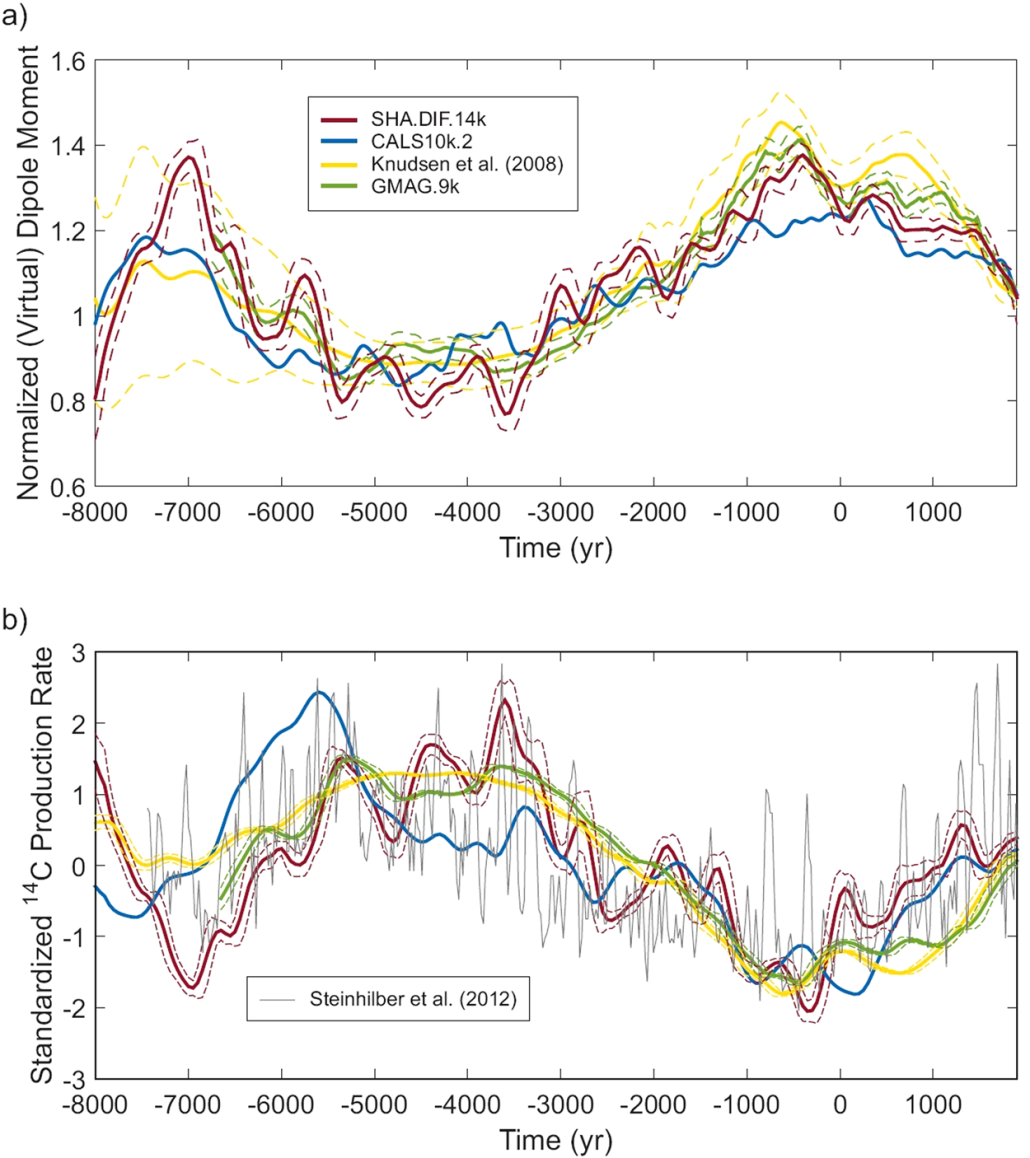
(**a**) The Earth’s magnetic dipole moment for the last 10ka according to different paleomagnetic reconstructions (see legend). Dashed lines correspond to 1σ of uncertainty (the CALS10k.2 model, blue line, does not provide error information). The dipole moment is normalized to the present value of 7.9·10^22^ A·m^2^. (**b**) Global average RcPR curves (red, blue, green and yellow lines, with 1σ of uncertainty in dashed lines) based on the paleomagnetic reconstructions displayed in (**a**) together with the original RcPR curve (grey line) of Steinhilber *et al*.^25^. All the curves are standardized for comparison, i.e. the corresponding mean is subtracted from each value of the curves. Then, these differences are divided by the standard deviation. The 1σ uncertainty in the curves based on paleomagnetic reconstructions is obtained by 5000 random iterations of the bootstrap approach taking into account the dipole moment and inclination (magnetic latitudes) uncertainties in the model of Masarik and Beer^3^.

To derive RcPRs, we use four types of paleomagnetic reconstructions covering the Holocene (see Fig. 1a): a) the average dipole moment of Knudsen *et al*.^14^; b) the average dipole estimations of Usoskin *et al*.^6^ –called GMAG.9k–; c) the spherical harmonic model CALS10k.2^19^, based on all kind of paleomagnetic data; and d) the spherical harmonic model SHA.DIF.14k^18^ based only on archeomagnetic and volcanic data. To link geomagnetic field variations with radiocarbon estimations, we apply the physical model of Masarik and Beer^3^. The solar contribution has been considered constant for the entire time interval and equal to the mean value for the Holocene (i.e. a solar modulation potential equal to 550 MV^10^). To obtain the radionuclide production changes from natural archives, the combined cosmogenic record of Steinhilber *et al*.^25^ has been used (Fig. 1b). This record combines different ^10^Be ice records from Greenland and Antarctica with the global ^14^C tree ring record and the principal component analysis is applied to eliminate the so-called system effects mitigating the high-frequency (periods shorter than 100 yr) noise present in individual radionuclide records^25^.

## Results

### On the role of magnetic harmonic contributions in the global average radionuclide production rate

As mentioned above, the Earth’s magnetic main field is defined by the sum of dipolar (degree *n* = 1, that corresponds to a tilted geocentric dipole field) and non-dipolar (degree *n* ≥ 2) harmonic contributions. Here, we analyse the influence of the different geomagnetic harmonic degrees *n* to the global average RcPRs. In the following, we present the results obtained using the SHA.DIF.14k model (in the supplementary material the results obtained with the CALS10k.2 model are also shown). Note that the paleomagnetic reconstructions of Knudsen *et al*.^14^ and Usoskin *et al*.^6^ –GMAG.9k– are not used in this section since they only contain the first harmonic degree, i.e. the dipole.

**Figure 2:**
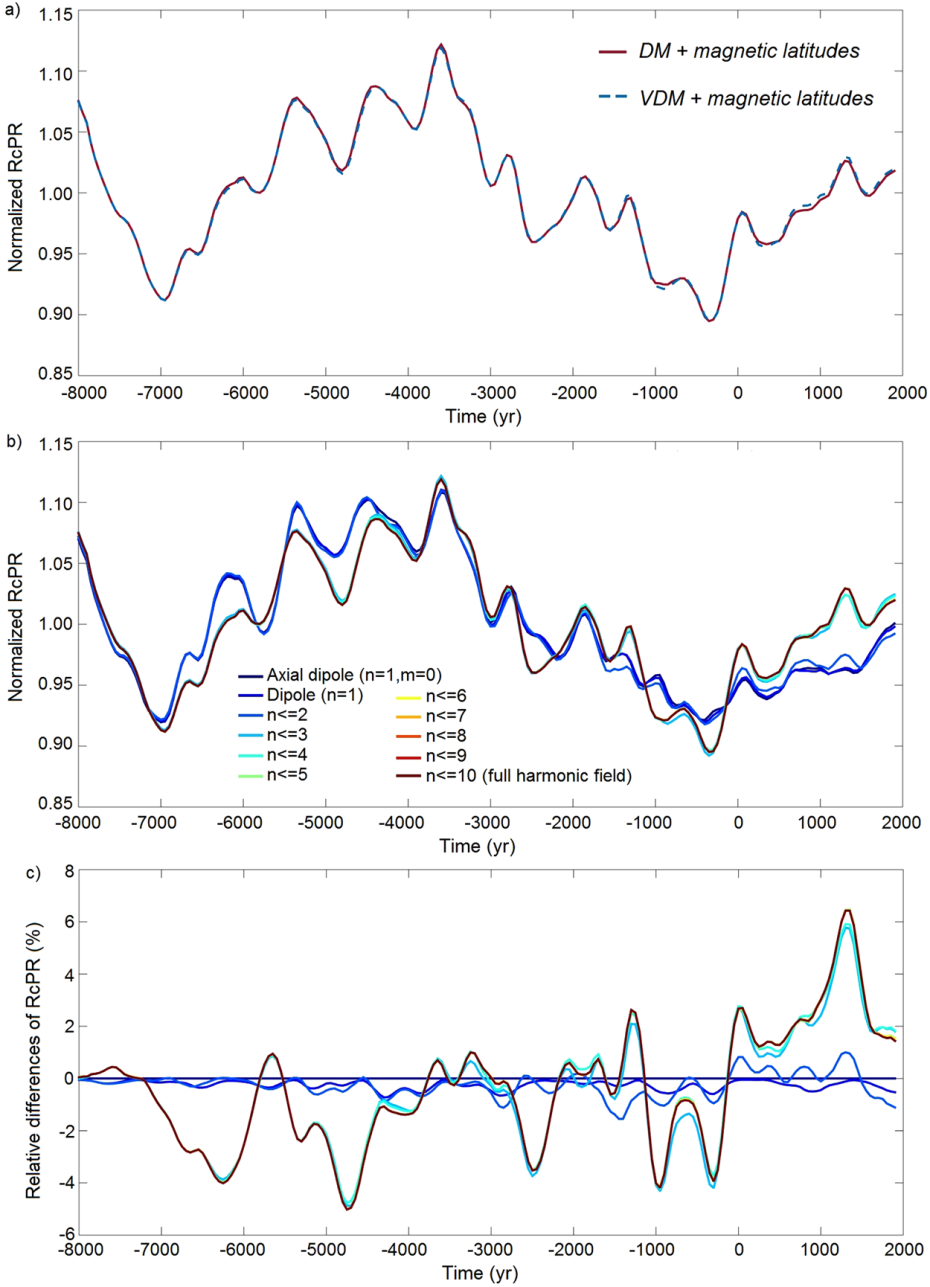
(**a**) Global average RcPR based on the SHA.DIF.14k geomagnetic model. Red line corresponds to the RcPR calculated when the dipole moment and local magnetic latitudes are considered in the Masarik and Beer^3^ model. Dashed blue line provides the global average production rate when both local virtual dipole moments and magnetic latitudes are used (see Methods). (**b**) Global average RcPR based on the SHA.DIF.14k geomagnetic model using the dipole moment and the local magnetic latitudes for a sum of increasing harmonic contributions (see legend) from the axial dipole field up to the 10-degree full harmonic field. (**c**) Relative differences of all the curves shown in (**b**) respect to that given by the axial dipole field (values are given in %). Same color code for the harmonic terms of (**b**). RcPR curves in (**a**) and (**b**) are normalized.

The model of Masarik and Beer^3^ involves two magnetic parameters to estimate the RcPR at local scale (before its global average): the strength magnetic field and the magnetic latitude (*ϕ*_*mag*_). The local strength field is represented by the so-called virtual dipole moment (VDM), which in turn depends on both the intensity and inclination values, and *ϕ*_*mag*_ is derived from the inclination (see Methods). In the upper troposphere the local inclination and intensity depend on the maximum harmonic degree *n* of the Earth’s magnetic field. Consequently, the RcPR is a function of the degree *n* for a given location *i*, i.e. *R*_i_(*n*) = *R*_*i*_[VDM(*n*),*ϕ*_*mag*_(*n*)]. However, previous studies^6,10,11^ have used the first degree approximation of the VDM, i.e. the dipole moment (DM), to represent the strength field at global scale (see Methods). In this particular case, the local function *R*_*i*_(*n*) can be rewritten as R*_i_(*n*) = R*_i_[DM, *ϕ*_*mag*_(*n*)]. Our first step is to evaluate the global average (denoted by < >) of both functions, i.e. *R*(*n*) = < *R*_*i*_(*n*) > and R*(*n*) = < R*_i_(*n*) > , in order to check if the use of the DM or the VDM in the Masarik and Beer^3^ model produces different results. To do that, we apply the SHA.DIF.14k model using the full degree, i.e. *n* = 10. Global averages *R*(*n*) and R*(*n*) are calculated every 50 yr from 8000 BC to 1900 AD getting two temporal curves for each case (see Methods for more details about this approach). Results, plotted in Fig. 2a, show that the time variation of *R*(10) and R*(10) are very similar. This agreement is independent of the chosen maximum degree *n*, as shown in the set of global average curves provided in Fig. 1S (i.e. *R*(*n*) ≅ R*(*n*) for a given time). These curves are obtained from the SHA.DIF.14k model by changing the maximum degree *n* from 1 (including the axial field) to 9. This result indicates that one can use directly the dipole moment as the representative strength field to evaluate the local RcPR before its global average.

However, *R*(*n*) depends on the degree *n*, i.e. *R*(*n*) *≠* *R*(*n’*) for *n* *≠* *n’*. The previous curves (Figs 2a and 1S) all together allow evaluating the dependence of the global average RcPR on the (maximum) degree *n*. For this purpose we plot in the same figure (Fig. 2b) all the average global curves of RcPR, *R*(*n*), for different maximum *n* (from degree 1 to 10). Results display two clear groups of curves depending on the maximum degree. For the axial (degree 1 and order 0), dipole (degree 1), and dipole + quadrupole (degrees 1 + 2), similar RcPRs curves are obtained, i.e. *R*(1) ≅ *R*(2). For higher degrees (i.e. the sum up to the octupole –degree 3–, and higher) all the curves are superposed following the same trend (*R*(3) ≅ *R*(4)≅ … ≅*R*(10)). Both groups of curves agree for long timescales, but exhibit different fluctuations at short periods. We estimate the relative differences between the curve obtained by the axial dipole field and those given by higher harmonic degrees. The relative differences (Fig. 2c) show a clear discrepancy with a maximum around ±7%. In order to check if this relative maximum is significant, we apply an error propagation scheme to infer error bands to the RcPR curves based on the SHA.DIF.14k uncertainties (see Methods). Figure 2Sa shows the results for the axial dipole, dipole, dipole + quadrupole, and the full 10-degree field. We conclude that the observed maximum relative differences of ±7% are significant since there is not overlapping between the curves when the error bands are considered. This is a novel and important result, since we demonstrate that the maximum harmonic degree of the paleomagnetic reconstruction plays an important role in the global average of RcPRs. For a correct application of a paleomagnetic field reconstruction in the modulation of radiocarbon rates at least the sum of the dipole, quadrupole and octupole field (i.e. the first 3 harmonic degrees) must be considered. On the contrary, some fluctuations of the paleomagnetic field could not be taken into account (see Discussion for more details).

Finally, it is worth mentioning that similar results are obtained if the harmonic model CALS10k.2 is considered (see Figs 2Sb and 3S). In addition, the alternative physical model of Kovaltsov *et al*.^4^ has been used instead of the Masarik and Beer^3^ model and similar values of global average of RcPRs are obtained (see Fig. 4Sa).

### Frequency-domain components of global average RcPR

In this section we analyse the frequency-domain of the global average RcPR curves derived from geomagnetic field reconstructions and we compare them with those derived from natural archives (see Fig. 1b) in order to identify patterns of covariability between both curves. Note that the two sources of information are not independent (since the Earth’s magnetic field modulates the RcPR) and hence, the existence of coherence in amplitude and phase between them could be interpreted as an evidence that the geomagnetic field drives specific scales of variability in isotope production.

Previous works, devoted to analyse the relationship between radionuclides production and the geomagnetic field have addressed this problem in a different way. They assumed that the long-term variability observed in radionuclide production curves is caused by the geomagnetic field and that the high frequency is related to solar magnetic activity. Then, they applied different low-pass filters to the natural signal of radionuclides records to estimate the past changes in the dipole moment to finally compare them with dipole moment reconstructions obtained from paleomagnetic data^6,10,11^. Here we apply an alternative approach, which consists on calculating the RcPR based on different paleomagnetic reconstructions and, then, the obtained results are compared with the independent original production curves of Steinhilber *et al*.^25^ derived from natural archives. Following the results explained in the previous section, we estimate the local RcPR using the dipole moment and the magnetic latitudes. Then, we calculate the global average curves of RcPR (see Methods). Results are plotted in Fig. 1b along with the curve of Steinhilber *et al*.^25^.

Logically, the RcPR curve based on the Knudsen *et al*.^14^ dipole moment presents the smoothest behaviour due to the large width of 500 yr (1000 yr for ages older than 4250 BC) of the averaging window used. The shorter width of 200 yr (500 yr for ages before 1500 BC) used for the GMAG.9k curve provides an increased variability. The higher variability observed when the spherical harmonic models SHA.DIF.14k or CALS10k.2 are used can be explained by two effects: a) the temporal parameterization applied and based on cubic B-splines with knot points every 40–50 yr; and b) the use of the magnetic latitudes instead of geographic latitudes in the radiocarbon modulation process. Fig. 1b also shows that the SHA.DIF.14k presents, in general, the same trend than previous global reconstructions but with a higher temporal variability. On the contrary, the CALS10k.2 curve presents clear temporal shifts in the depicted maxima and minima with respect to the other curves. These shifts can be traced back to the use of sedimentary data during the modelling approach.

In order to characterize the frequency content of the geomagnetically-based curves and compare it with the results for the original radionuclide production curve we apply several analyses in the frequency-domain (see Methods). Firstly, a spectral analysis by means of the continuous wavelet and the classical Fourier transform is applied to identify the dominant timescales of variability. Secondly, we carry out a cross-spectrum study to analyse the coherence and delay between the different curves. Finally, we apply the empirical mode decomposition (EMD) to isolate the intrinsic mode functions that compose the original signal. In all the cases, a Monte-Carlo bootstrap approach is applied to estimate uncertainties (see Methods for more details) providing more robustness to our results.

**Figure 3:**
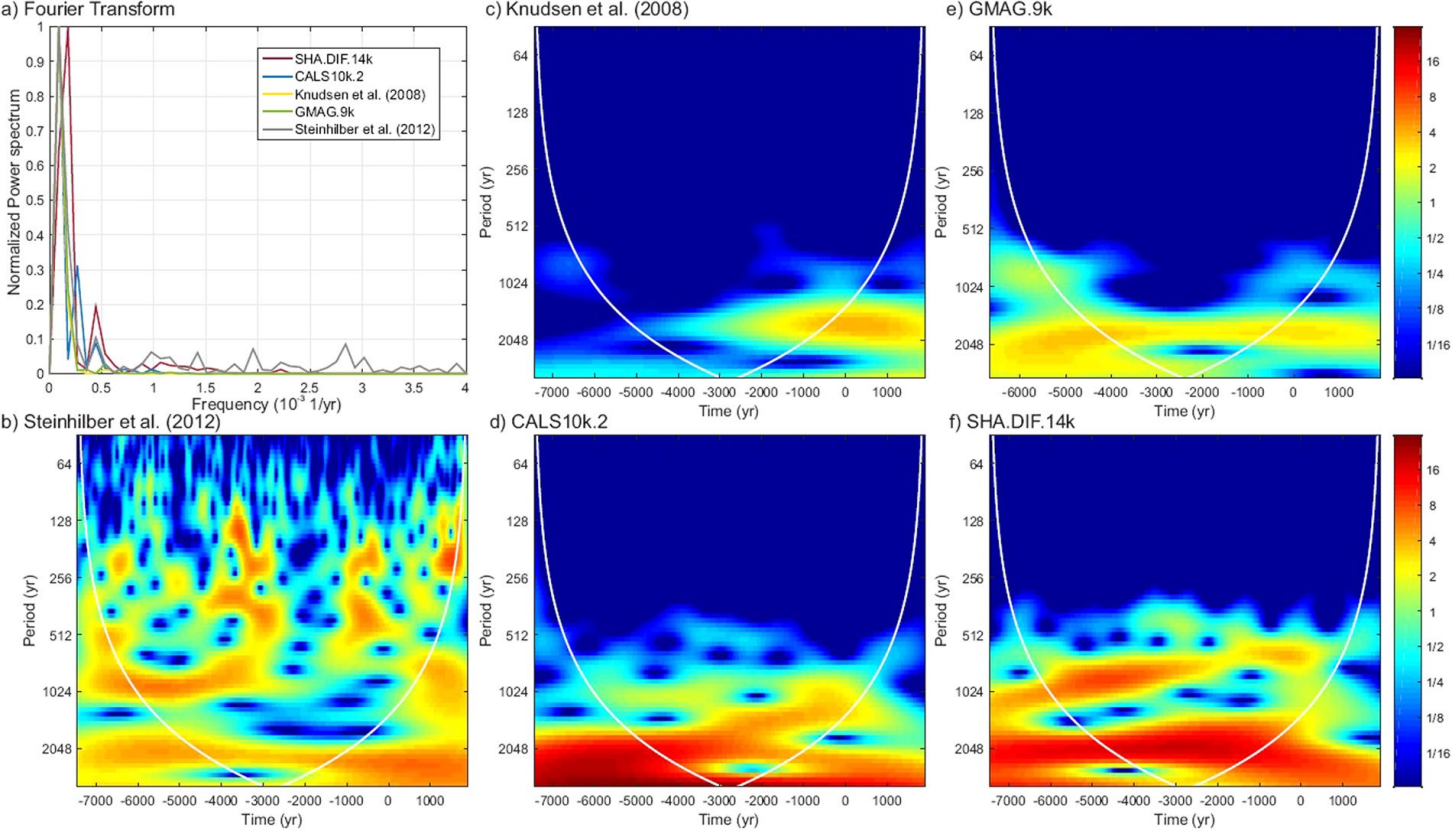
Frequency-domain analysis. (**a**) Fourier transform for the different radionuclide production rate curves (see legend). For each curve, the amplitude of the power spectrum is normalized to the maximum value. (**b**–**f**) Wavelet analysis with a Morlet basis functions for the radionuclide production rate curves. For this calculation only the mean value of the radiocarbon production rates (Fig. 1b) are used.

The Fourier transform shows the range of the characteristic frequencies obtained for the different curves (Fig. 3a). The original radionuclide curve covers an expanded range of frequencies as expected due to its high temporal variability, with significant peaks in the power spectrum around ∼0.18·10^−3^, ∼0.44·10^−3^, ∼1.2·10^−3^, ∼1.42·10^−3^, ∼1.95·10^−3^, ∼2.84·10^−3^ yr^−1^ (periods of ∼6000, ∼2400, ∼850, ∼700, ∼500, and ∼350 yr). The power spectrum obtained from the paleomagnetic curves (Fig. 3a) presents only two significant frequencies, 0.18·10^−3^ and 0.44·10^−3^ yr^−1^ (periods of ∼6000 and ∼2200 yr), these being in close agreement with the lowest frequencies of the radionuclide curve. The CALS10k.2 curve also shows a maximum at ∼0.30·10^−3^ yr^−1^ (3500 yr). The curve based on the SHA.DIF.14k model presents an additional significant range of frequencies around 1.10·10^−3^ – 1.25·10^−3^ yr^−1^ (800–900 yr) and 2.2·10^−3^ yr^−1^ (500 yr), in agreement with the radionuclide results. No significant frequencies above 2.2·10^−3^ yr^−1^ (periods lower than 500 yr) are found in the curves of geomagnetic origin.

The wavelet analysis (see Methods) for each curve shows that the comparatively smoothed Knudsen *et al*.^14^ curve presents a significant timescale of variability centred at ∼2000 yr since ∼3000 BC (Fig. 3c) that can also be found in the GMAG.9k curve (Fig. 3e), for the entire time interval (6750BC–1900 AD). The GMAG.9k curve also shows a shorter period of about 800–900 yr before 4500 BC and after 1000 BC. The CALS10k.2 and SHA.DIF.14k reconstructions (Fig. 3d,f) present a higher variability with characteristic periods of about 2200 yr. The SHA.DIF.14k curve shows, in addition, a significant variability at 800–900 yr. The original radionuclide record (Fig. 3b) points out the highest temporal variability with a wide range of significant periods. However, two dominant timescales centred at ∼2200 and ∼800 yr can be clearly identified. These periods are similar to the wavelet results obtained with the SHA.DIF.14k model. Importantly, the other geomagnetic curves do not provide the significant period of ∼800 yr for the whole time interval.

**Figure 4:**
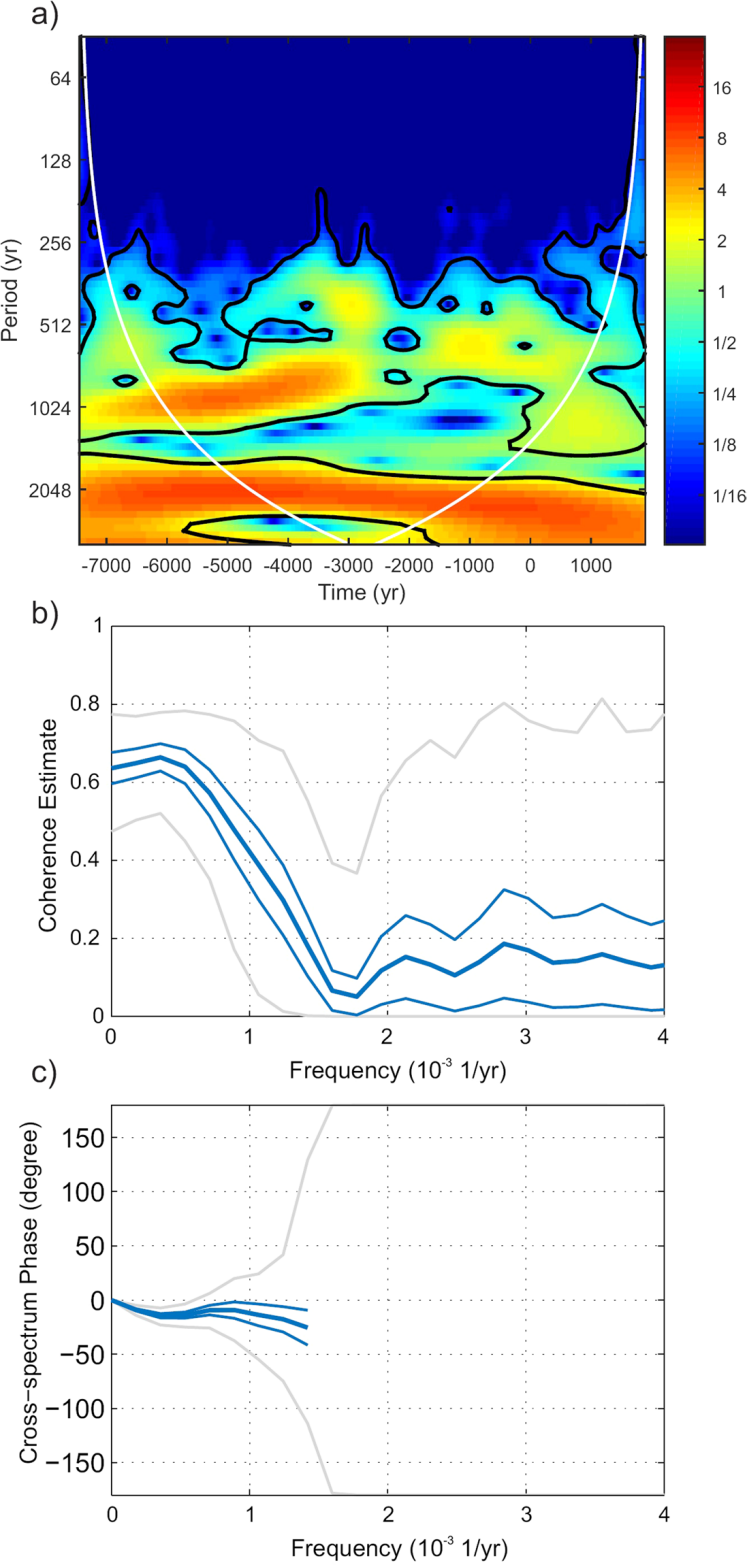
Correlation analysis between the original production rate curve obtained from natural archives^25^ and production rates derived from the SHA.DIF.14k geomagnetic field model. (**a**) Correlation wavelet analysis using Morlet basis functions. Black lines show significant correlations (α < 0.05). (**b**) Coherence between both curves based on the magnitude-square coherence function (see Methods). This function ranges between 0 (without coherence) and 1 (perfect coherence). (**c**) Phase lag between both curves according to the cross-spectrum analysis. In (**b**) and (**c**), thick blue line represents the mean values obtained after applying the bootstrap approach and the thin blue lines the 1σ uncertainty. The gray lines in (**b**) and (**c**) are the envelope total bootstrap iterations. In (**c**) the error bar at 1σ has been plotted only from 0 to 1.25·10^−3^ yr^−1^ since after this frequency the cross-spectrum phases take random values between −180 and 180 degrees.

To further investigate the similarity between the original and the SHA.DIF.14k curves, we carry out a cross correlation wavelet analysis (Fig. 4a). Results show that both curves share signals of ∼2200 yr and ∼800 yr (α < 0.05). In addition, the coherence estimation (Fig. 4b) between both curves also corroborates these common frequencies, with values of the magnitude-square coherence higher than 0.4 for frequencies lower than ∼1.25·10^−3^ yr^−1^ (periods higher than 800 yr) and 0 degrees of delay as shown in the cross-spectrum phase diagram (Fig. 4c). To estimate the uncertainty in the correlation analysis performed, the 1σ error of the curves has been taking into account by using the bootstrap approach (see Methods). Results are plotted in Fig. 4b,c along with the total envelope of Monte-Carlo iterations. In Fig. 4c the error bar at 1σ has been plotted only from 0 to 1.25·10^−3^ yr^−1^ since the cross-spectrum phases show random values between −180 degrees and 180 degrees after this frequency.

**Figure 5:**
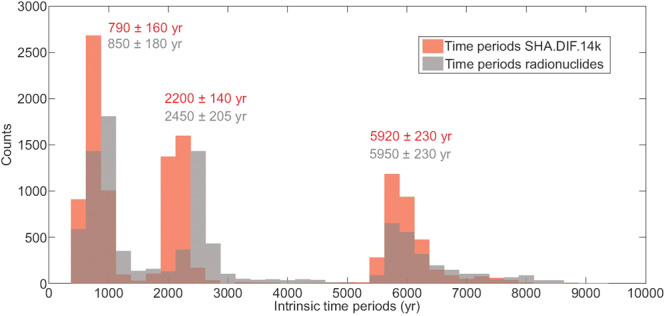
Histograms showing the characteristic or intrinsic periods of the Steinhilber *et al*.’s curve (grey bars) and the SHA.DIF.14k production rate curve (red bars). Periods are obtained by the bootstrap random iterations. Periods lower than 500 years are removed (see text for details). The mean value of the different periods and their 1σ are also indicated in the figure.

To better characterize the common periods of both signals, we apply the EMD method taking into account the bootstrap procedure. The results show three groups of intrinsic periods for the two studied curves (see Fig. 5), corresponding to 5950 ± 230 yr, 2450 ± 205 yr and 850 ± 180 yr for the radionuclide results and 5920 ± 230 yr, 2200 ± 140 yr, and 790 ± 160 yr for the SHA.DIF.14k curve. The intrinsic periods lower than 500 yr identified in the RcPR curve (i.e. 375, 195 and 110 yr) were not considered since they are not significant in the geomagnetic curve.

**Figure 6:**
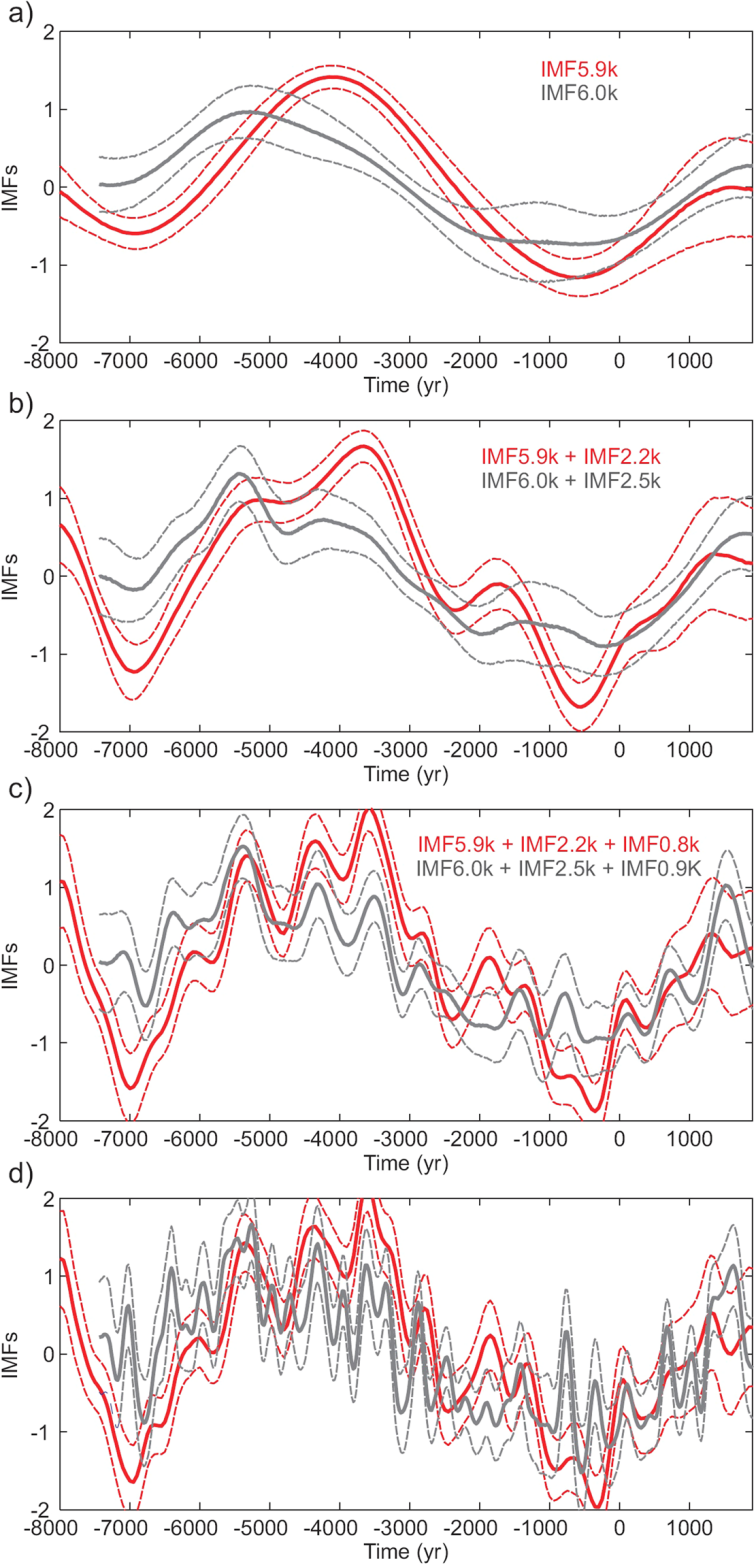
Intrinsic mode functions (IMF) for the production rate curves (in red the one based on the SHA.DIF.14k model and in grey the original curve based on natural archives). (**a**) The last intrinsic mode (largest period). (**b**) Sum of the last two modes. (**c**) Sum of the last three modes. (**d**) Sum of the last four modes.

Finally, we decompose both time series into the Intrinsic Mode Functions (IMF) (Fig. 6) starting from the last mode (i.e. the largest period, Fig. 6a) and then adding the IMF with lower characteristic periods. The sum of the second (Fig. 6b) and third (Fig. 6c) consecutive IMF shows a similar behaviour for both time series in terms of amplitude and phase, with a maximum correlation for the sum of the last three IMF (Fig. 6c). The observed coherence disappears when we add the next IMF with a lower intrinsic period (∼375 yr; Fig. 6d).

It is noteworthy that very similar results are obtained if the alternative radiocarbon production curve proposed by Roth and Joos^26^ is used. Figure 4Sb shows the application of the EMD method to this curve. It can be seen that the same range of dominant periods are obtained.

## Discussion

**Figure 7:**
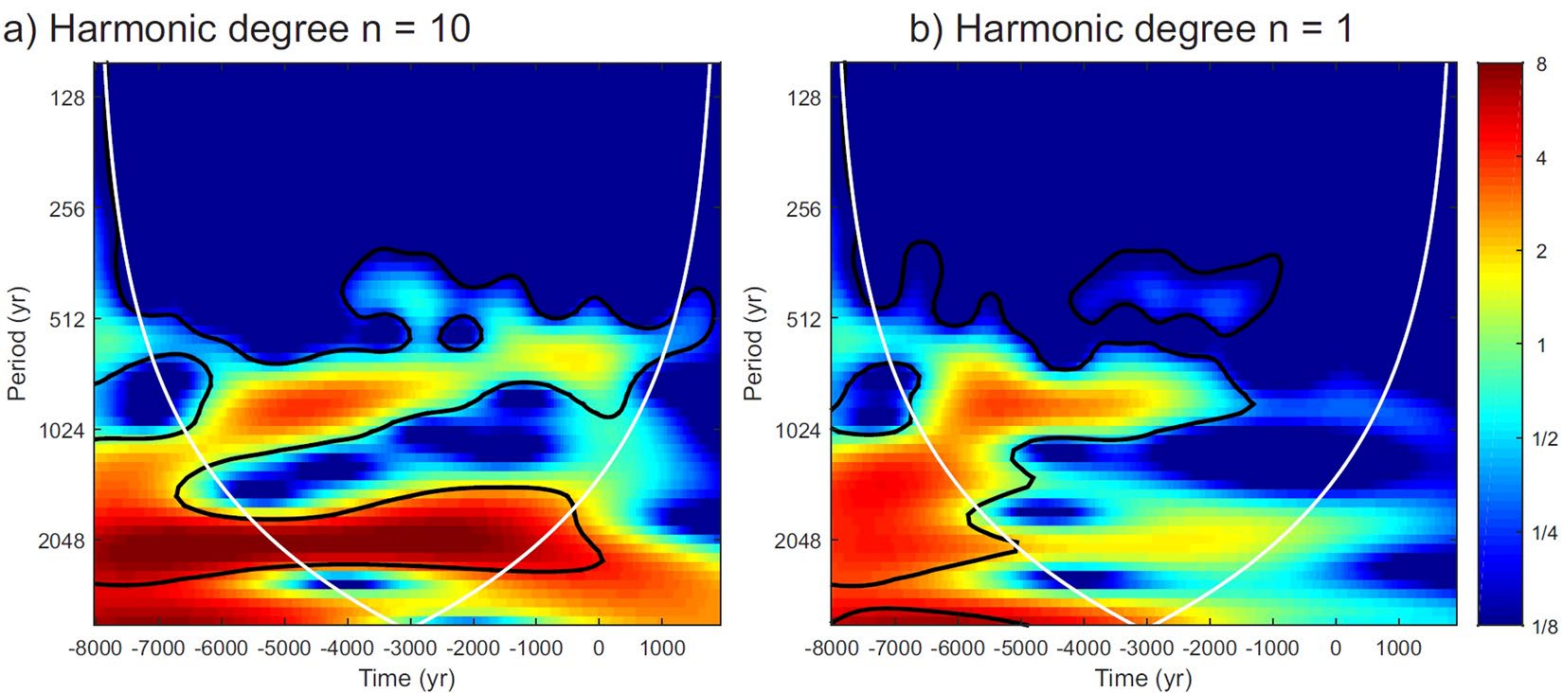
Wavelet analysis with a Morlet basis functions for the two different RcPR curves. (**a**) RcPR based on the full harmonic SHA.DIF.14k model (degree *n* = 10). (**b**) RcPR based on the SHA.DIF.14k model considering only the dipole field (harmonic degree *n* = 1).

The results exposed before provide a new perspective of the geomagnetic field modulation on the production rates of cosmogenic radionuclides in the atmosphere. Our results demonstrate that, during the Holocene, the global average rate is not only controlled by the strength of the geomagnetic field as it is given by the dipole moment but also that changes in the non-dipole harmonic degrees exert an important role. In previous studies related to this topic, it has been assumed that the effects of the non-dipole degrees are negligible. Here we invalidate this common hypothesis by demonstrating that maximum differences of ∼7% in the global average RcPR are obtained when the geomagnetic field at least up to harmonic degree three (i.e. sum of the dipole, quadrupole and octupole) is considered. Moreover, several discrepancies are also observed in the time-frequency domain. The observed periods of ∼800 yr and ∼2200 yr are present throughout the total time interval when harmonic degrees higher than 3 are considered, i.e. the octupole (see the wavelet analysis of Fig. 7a), whereas they are not significant for the total time window for the RcPR curve based on the first two harmonic degrees (see Fig. 7b). From this analysis, we can deduce that the effect of non-dipolar terms is significantly larger than generally assumed.

It was recently suggested that the characteristic period of ~2400 yr found in radionuclides production curves (the so-called Hallstatt cycle) is linked to the long-term variability of solar activity^6^. These authors conclude that this period has a solar origin based on two assumptions: a) the radiocarbon mixture occurs at a global scale and then is insensitive to the geomagnetic migration of the poles; and b) the observed radionuclide production rates from natural archives should be associated to a dipole variation of 2·10^22^ A·m^2^ and this is not supported by any paleomagnetic reconstruction. On the contrary, our analysis support the hypothesis that changes in the strength and magnetic latitudes given by at least 3-degree harmonic models during the Holocene (as computed by state-of-the art global spherical harmonic geomagnetic models) can be the primary cause of the Hallstatt cycle. The importance of geomagnetic tilt was already suggested in the study of Vasiliev *et al*.^27^. Our results go beyond this hypothesis, and suggest that the Hallstatt cycle has most likely a geomagnetic origin and, in particular, is associated to the geomagnetic field when it is considered at least as the sum of the first three harmonic degrees. These results do not exclude that solar activity could also present a ~2400-yr period, but this issue is beyond the scope of this work.

Therefore, the approaches (commonly used up to now) of applying the models of Masarik and Beer^3^ or Kovaltsov *et al*.^4^ considering only the dipole moment without magnetic latitude information (see e.g. Fig. 1 in Snowball and Muscheler^8^ and Fig. 2 and Table of Supplementary Material of Kovaltsov *et al*.^4^) should be revised. In addition, dipole moment reconstructions based on global averages of paleointensity data and thus not containing information about past magnetic latitudes^6,14^ do not provide the best approach to estimate past changes in solar activity. The new approach presented here requires the use of the recently developed spherical harmonic geomagnetic models, which provide a powerful way to synthetize the past variations of the dipole moment strength together with magnetic latitude changes. This is, as demonstrated before, the best approach to consider and correctly evaluate the contribution of geomagnetic field changes on radionuclide production rates.

Moreover, our results demonstrate that a robust correlation, in terms of amplitude and phase, exists between the original curve of the radionuclide production^25^ and the curve synthesized from the SHA.DIF.14k geomagnetic field model. Three ranges of time periods: 790–850 yr, 2200–2450 yr, and 5920–5950 yr, have been revealed in both curves at 95% of probability (Fig. 5). The correlation with the original radionuclide curve is also observed when the other paleomagnetic reconstructions are used, but with a lower degree of robustness. These results suggest that the geomagnetic field modulates radionuclide production in the atmosphere not only at the millennial scale (periods of 2200 and 6000 yr), but also at the centennial time-scale (period of 800 yr). This statement is supported by a clear coherence in terms of amplitudes and phases between the original and geomagnetically derived radionuclide productions and revealed using different frequency-domain analysis techniques (Fourier transform, wavelets, EMD).

This has potential important implications in our present knowledge of the solar activity. The two dominant periods detected in the geomagnetic activity (of about 800 and 2200 yr) are not currently being considered in the three-dimensional relationship between radionuclides production rates, geomagnetic field variations and solar activity. Therefore, multi-centennial fluctuations in radionuclide production are only being ascribed to past solar activity changes.

**Figure 8:**
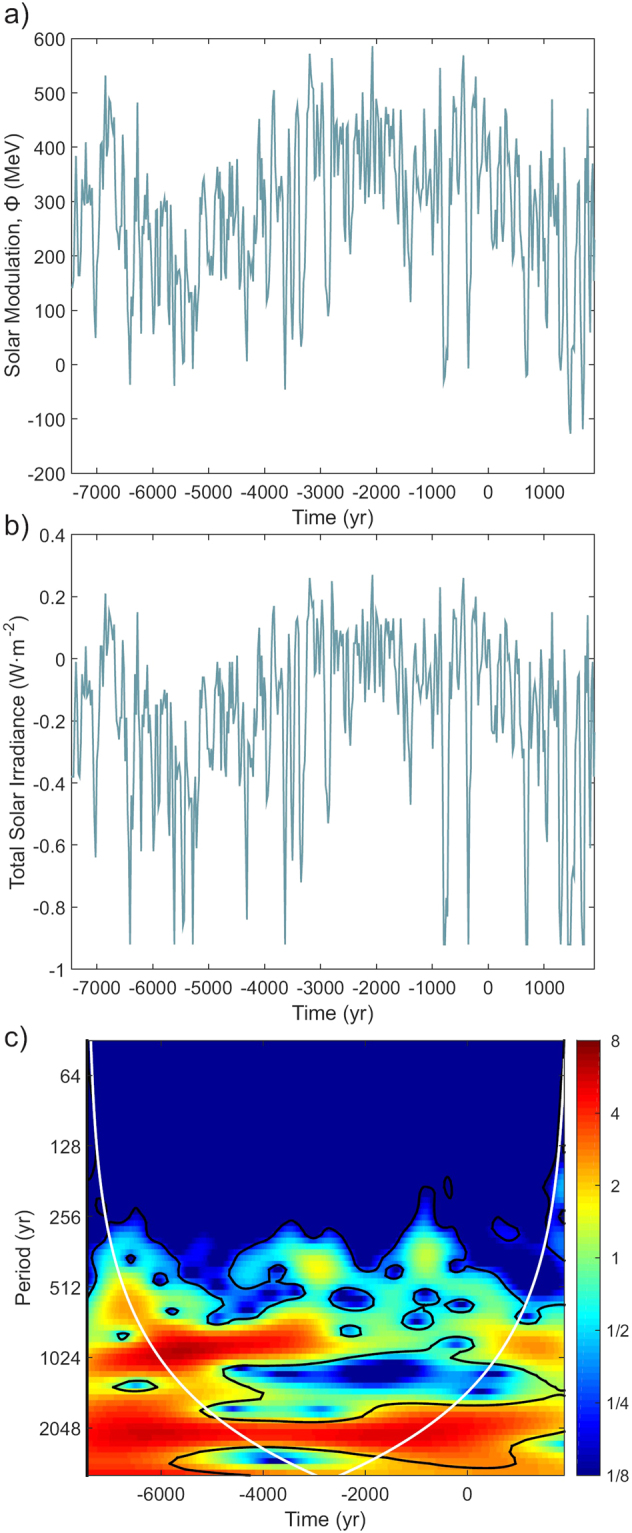
(**a**) Solar activity by means of the solar modulation potential estimated by Steinhilber *et al*.^25^. (**b**) Total solar irradiance, TSI, derived from the solar modulation potential in (**a**). (**c**) Correlation wavelet analysis using Morlet basis functions between the TSI and the production rate based on the dipole moment and inclinations from the SHA.DIF.14 k model. Black lines show significant correlations (α < 0.05).

To further demonstrate this fact, we compare the frequency content of the reconstructed solar activity for the Holocene and the geomagnetic field evolution synthetized with the SHA.DIF.14k global model. The geomagnetic signal depends on the chaotic evolution of the geodynamic process that occurs in the Earth’s outer core; and the solar activity is associated to the Sun’s magnetic field that is governed by the turbulent magnetohydrodynamic processes of this star. A priori, and if the geomagnetic and solar modulation are properly disentangled both signals should be independent. To assess this independence, we use the solar activity reconstruction of Steinhilber *et al*.^25^ based on the physical model of Masarik and Beer^3^. In the Steinhilber *et al*.^25^ work the dipole moment of Knudsen *et al*.^14^ was used to estimate the past geomagnetic field modulation on cosmogenic isotopes production rates. The solar modulation potential Φ obtained by these authors is represented in Fig. 8a and the total solar irradiance (TSI) generated from Φ is plotted in Fig. 8b. The wavelet coherence analysis performed here between the Φ/TSI curve and the geomagnetic signal (dipole moment and inclination) is shown in Fig. 8c. The correlation is significant (α < 0.05) for timescales of ∼800 and ∼2200 yr over the entire studied time interval. Thus, both records are not independent.

In conclusion, at present, the effect of the Earth’s magnetic field on the radionuclide production rates in the atmosphere is not being properly estimated. This has important implications in our current knowledge of past solar activity, and consequently of the TSI. This should motivate the investigation of the possible geomagnetic field modulation at multi-centennial and centennial time-scales in order to better ascertain the past variability of solar activity. This requires the implementation of more precise spherical harmonic geomagnetic models and therefore, the availability of new paleomagnetic data for the Holocene. Moreover, this result challenges past and currently ongoing approaches to simulate past climate under the umbrella of the Paleoclimate Model Intercomparison Proyect, Phases 3 and 4 (PMIP3,4^22–24^). Within the PMIP frame, paleoclimate simulation experiments are planned using solar, volcanic and other natural and anthropogenic forcings as boundary conditions for transient climate experiments within the mid and late Holocene^1^. On the basis of the results presented herein, the solar forcing reconstructions used in PMIP3,4 experiments are, with high confidence, prone to be contaminated with geomagnetic field variability, thus influencing with an spurious signal climate model simulations. Such contamination can have also consequences for climate model-proxy data comparison exercises and for our understanding of solar-climate relationships within the affected timescale intervals.

## Methods

### RcPR based on paleomagnetic reconstructions

The Earth’s magnetic field is given by the sum of dipolar and non-dipolar harmonic contributions. Both terms decrease with altitude by the factor [*a*/(*a* + *h*)]^n+2^, where *a* is the mean Earth’s radius and *h* the altitude. According to Masarik and Beer^3^, the production of radiocarbon, due to cosmic ray interactions, occurs in the upper troposphere and lower stratosphere (∼10–20 km altitude) and, hence, this altitude is not sufficient to consider negligible the non-dipolar terms (i.e. degrees with n ≥ 2). For this reason, we evaluate here the influence of the geomagnetic harmonic contribution in the RcPR process.

For a constant solar modulation potential, Masarik and Beer^3^ consider the local radiocarbon production rate (before its mixing at global scale) as a function of the magnetic strength and the magnetic latitude (see Fig. 5S, modified from Masarik and Beer^3^). The magnetic strength can be represented at global scale by the dipole moment, *DM*, and at local scale by the virtual dipole moment, *VDM*^15^. The dipole moment is given by the first three Gauss harmonic coefficients (*g*_*1*_^*0*^*, g*_*1*_^*1*^*, h*_*1*_^*1*^) of the paleomagnetic reconstructions,

1 $$DM=\frac{4\pi {a}^{3}}{{\mu }_{0}}\sqrt{{({g}_{1}^{0})}^{2}+{({g}_{1}^{1})}^{2}+{({h}_{1}^{1})}^{2}}$$

where ${\mu }_{0}$ is the permeability constant. The *VDM* depends on the local magnetic intensity *F* and inclination *I*^15^ as follows:

2 $$VDM=\frac{2\pi {a}^{3}}{{\mu }_{0}}F\sqrt{1+3co{s}^{2}I}$$

It is worth to note that the DM represents the dipole field and it is a constant parameter for any location of the Earth, whereas the VDM contains information about higher harmonic degrees since the intensity and inclination elements depend on them. On the other hand, the local magnetic latitude, ${\varphi }_{mag}$, can be estimated by the magnetic inclination^28^:

3 $${\varphi }_{mag}=atan(\frac{1}{2}tan\,I)$$

when the dipole field is considered (degree *n* = 1), the magnetic latitudes in eq. [3] are denoted as geomagnetic latitudes. And these correspond to the geographic latitudes when the axial dipole field (degree *n* = 1 and order *m* = 0) is used.

The first step in the RcPR estimation is to calculate the parameters of eqs [1], [2] and [3] from paleomagnetic reconstructions. The *DM* is a global parameter, but the *VDM* and the magnetic latitudes must be synthetized at the local scale. To do that, we fix 1000 equally-distributed points over the Earth’s surface with a constant distance between neighbouring points of around 6.6° or 730 km. At each location, the inclination and intensity values are synthetized using the SHA.DIF.14k and CALS10k.2 models. We then apply the model of Masarik and Beer^3^ using the pairs “*DM*-magnetic latitude” or “*VDM*-magnetic latitude” to estimate the local radiocarbon production rate (using a constant solar modulation potential of 550 MV). Then, the global average RcPR is estimated as the mean value of the results obtained for the 1000 studied locations. Finally, the same procedure is performed for the rest of the temporal knot-points (every 50 yr) of the paleomagnetic harmonic reconstructions.

Note that for the case of Knudsen *et al*.^14^ and GMAG.9k curves, we assume that these curves represent axial *DM* estimations (only take into account the degree 1, order 0) and consequently, the magnetic latitudes correspond to geographic latitudes. In addition, the magnetic elements from both the SHA.DIF.14k and CALS10k.2 models were averaged in time using windows of 250 yr shifted every 50 yr, from 8000 BC to 1900 AD, to remove unreal high frequencies due to the paleomagnetic modelling process.

In order to calculate global average RcPR uncertainties, we consider the uncertainties derived from the paleomagnetic reconstruction to apply a Monte-Carlo bootstrap method, which takes into account the error bands of the different magnetic series. Following this procedure we obtain 5000 random curves of global average RcPR that provide the mean curve and its standard deviation. For the CALS10k.2 model, the bootstrap was not applied since this model does not provide uncertainties.

### Frequency-domain analysis of RcPRs

The different frequency analysis tools used in this work are detailed as follows. For each curve, the frequency content is analysed by the periodogram power spectra density (PSD) estimation. This tool uses the discrete Fourier transform to highlight the dominant frequencies of the studied signals. In addition, we carry out a wavelet spectrum study based on Morlet basis functions that quantify the frequency content of each signal during the total analysed time interval. The wavelet tool also provides the correlation of two curves by means of the wavelet coherence approach.

In order to evaluate the spectral coherence between two time-dependent curves, we apply the magnitude-square coherence function. This function, that depends on the PSD at each time series and the cross-PSD of both series, ranges between 0 (without coherence) and 1 (perfect coherence) and indicates how well the first time series corresponds to the second one for a particular frequency. In our study we use a minimum threshold of 0.4 to establish the coherence of both time series. The previous method is complemented by a cross-spectrum analysis that allows quantifying the phase lag (in degrees) between the two time series for the range of frequencies. The cross-spectrum phase ranges between −180 degrees and 180 degrees, where 0 degrees represents no time lag between the signals.

To decompose the original time series in their dominant frequencies, the empirical mode decomposition (EMD) tool is applied^29,30^. The approach decomposes time series into their intrinsic mode functions (IMF) represented by simple harmonic functions with characteristic instantaneous frequencies. The first IMF contains the highest frequency (shortest time period) and the last IMF presents the lowest frequency (larger time period). The sum of the different IMF´s (and the residual) reconstitutes the original signal.

Finally, the robustness of our results is highlighted by applying a Monte-Carlo bootstrap method, which takes into account the error bands of the different time series. To do that, we pick up every 50 yr a random value from the Gaussian distribution that represents the time series at that time. Following this procedure we obtain 5000 random curves providing to our results an error band at 1σ of probability.

## Electronic supplementary material

Supplementary Material
