# Author Correction: Multi-centennial fluctuations of radionuclide production rates are modulated by the Earth’s magnetic field

**DOI:** 10.1038/s41598-018-36666-9

**Published:** 2019-03-06

**Authors:** F. J. Pavón-Carrasco, M. Gómez-Paccard, S. A. Campuzano, J. F. González-Rouco, M. L. Osete

**Affiliations:** 1 Universidad Complutense de Madrid, 28040 Madrid, Spain; 2 Instituto de Geociencias IGEO (UCM-CSIC), 28040 Madrid, Spain; 3 Present Address: Istituto Nazionale di Geofisica e Vulcanologia (INGV), 00143 Rome, Italy

Correction to: *Scientific Reports* 10.1038/s41598-018-28115-4, published online 29 June 2018

This Article contains errors in the Discussion section.

“On the basis of the results presented herein, the solar forcing reconstructions used in PMIP3,4 experiments are, with high confidence, prone to be contaminated with geomagnetic field variability, thus influencing with an spurious signal climate model simulations.”

should read:

“On the basis of the results presented herein, climate model simulations using solar forcing reconstructions corrected by an axial dipole geomagnetic field are prone to be contaminated with geomagnetic field variability.”

