# Supplementary material for: Multi-centennial fluctuations of radionuclide production rates are modulated by the Earth’s magnetic field
Source: Sci Rep. 2018 Jun 29;8:9820. doi: 10.1038/s41598-018-28115-4 (PMC6026124; doi:10.1038/s41598-018-28115-4)

Supplementary Material

Multi-centennial fluctuations of radionuclide production rates are modulated by the Earth's magnetic field.

Pavón-Carrasco, F.J.[1,2*], Gómez-Paccard, M.[2], Campuzano, S.A.[1,2♣], González-Rouco, J.F.[1,2], Osete, M.L.[1,2]

[1] Universidad Complutense de Madrid, 28040 – Madrid, Spain.

[2] Instituto de Geociencias IGEO (UCM, CSIC), 28040 – Madrid, Spain.

* Corresponding author: fjpavon@ucm.es. Facultad de CC. Físicas. Universidad Complutense de Madrid. Avda. Complutense s/n. 28040 – Madrid (Spain).

♣Now at Istituto Nazionale di Geofisica e Vulcanologia (INGV), 00143 – Roma, Italy.

Abstract

The production of cosmogenic isotopes offers a unique way to reconstruct solar activity during the Holocene. It is influenced by both the solar and Earth magnetic fields and thus their combined effect needs to be disentangled to infer past solar irradiance. Nowadays, it is assumed that the long-term variations of cosmogenic production are modulated by the geomagnetic field and that the solar field dominates over shorter wavelengths. In this process, the effects of the non-dipolar terms of the geomagnetic field are considered negligible.

Here we analyse these assumptions and demonstrate that, for a constant solar modulation potential, the geomagnetic field exerts a strong modulation of multi-centennial to millennial wavelengths (periods of 800 and 2200 yr). Moreover, we demonstrate that the non-dipole terms derived from the harmonic degree 3 and above produce maximum differences of 7% in the global average radiocarbon production rate. The results are supported by the identification, for the first time, of a robust coherence between the production rates independently estimated from geomagnetic reconstructions and that inferred from natural archives. This implies the need to review past solar forcing reconstructions, with important implications both for the assessment of solar-climate relationships as well as for the present and future generation of paleoclimate models.

Figure 1S. Global average RcPR based on the SHA.DIF.14k geomagnetic model. Red line corresponds to the RcPR calculated when the dipole moment and local magnetic latitudes are considered in the Masarik and Beer[4] model. Dashed blue line provides the global average production rate when both local virtual dipole moments and magnetic latitudes are used (see Methods). The different captions correspond to the increasing sum of harmonic contributions from the axial dipole to the harmonic degree 9. All curves have been normalized by the mean value.

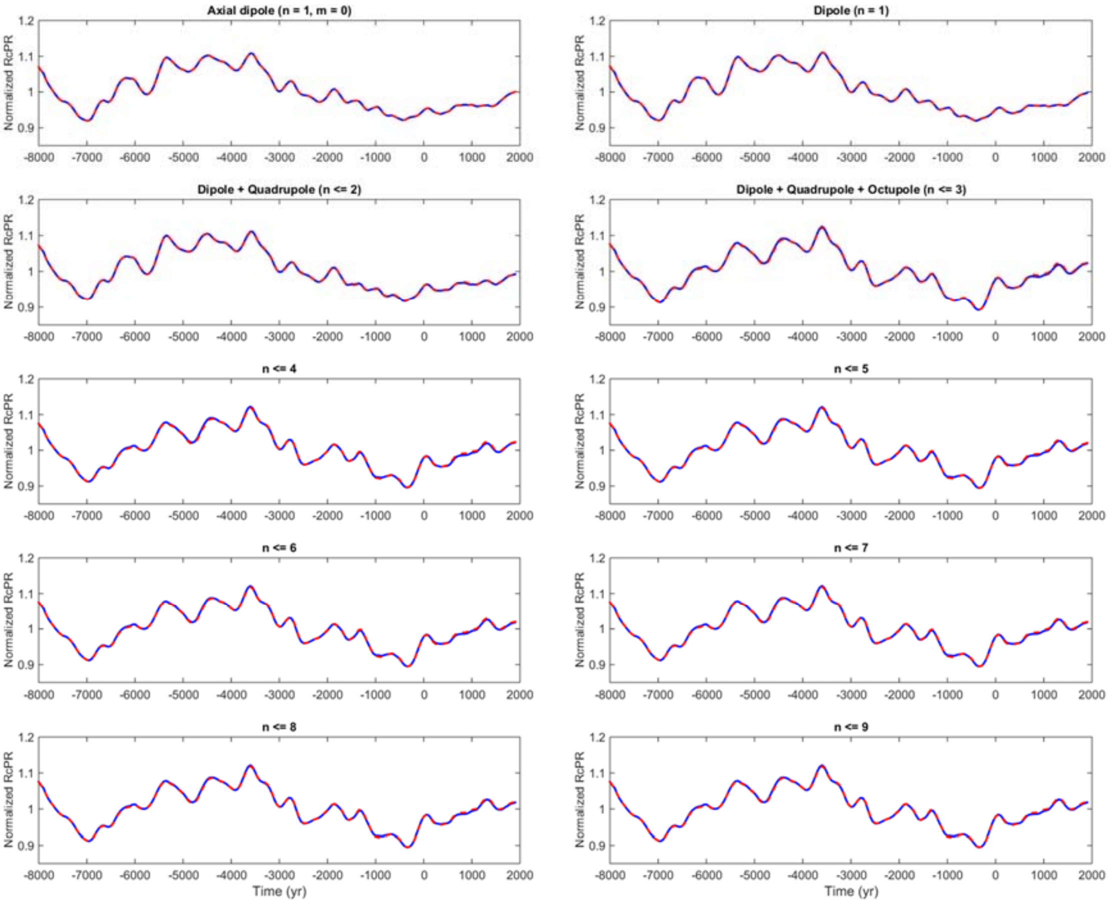

Figure 2S. Global average of the RcPR based on the a) SHA.DIF.14k and b) CALS10k.2 models using different maximum harmonic degrees (see legend). The error bands at 1-sigma are obtained by the bootstrap technique (see Methods). For the case of the SHA.DIF.14k model, the 1-sigma deviation also depends on the error of the magnetic latitudes (or inclinations) and dipole moment (or intensities). This is not the case of the CALS10k.2 since model errors are not available. This explains why the 1-sigma bands in CALS10k.2 are narrower than those given by the SHA.DIF.14k.

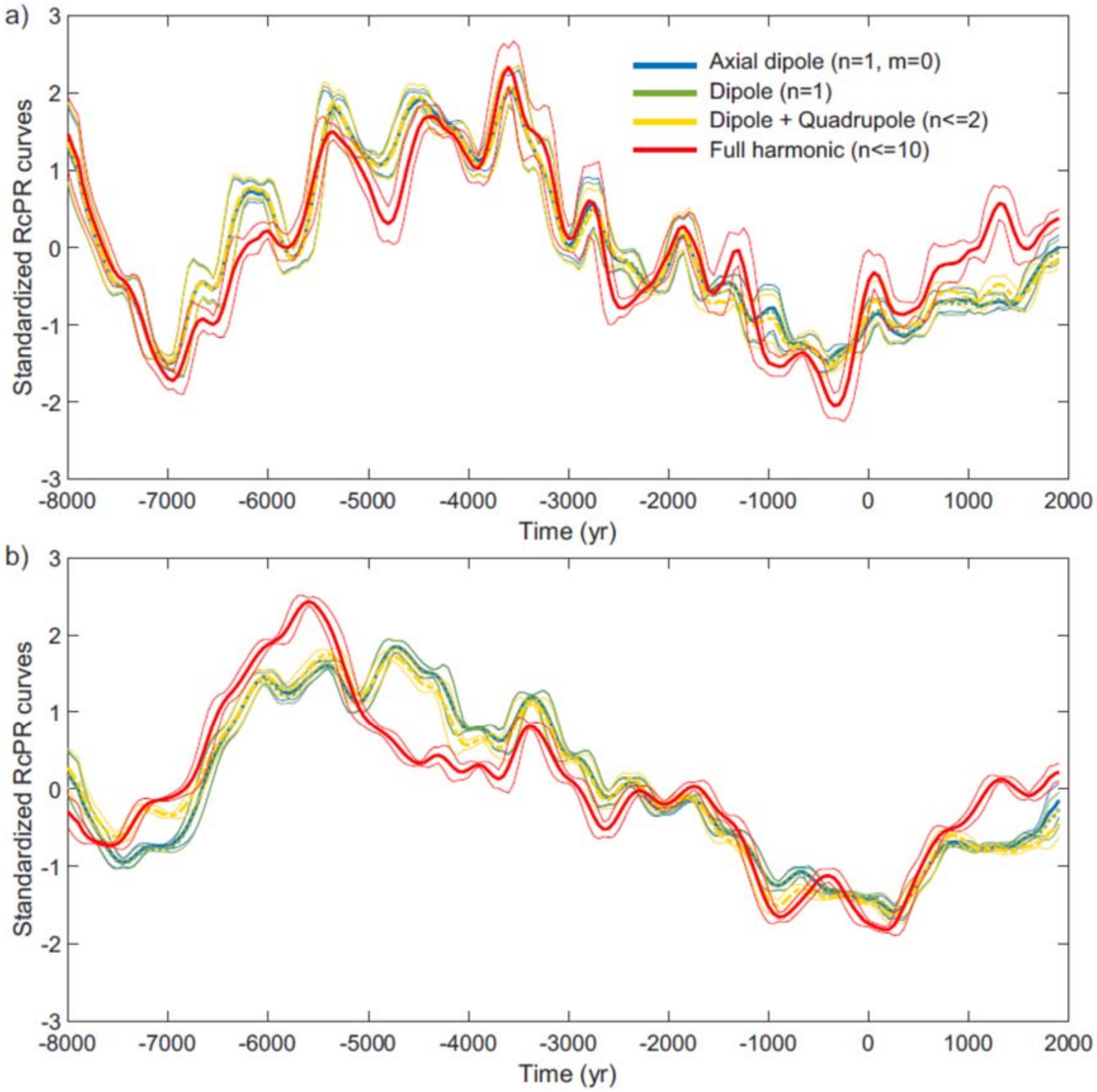

Figure 3S. a) Global average RcPR based on the CALS10k.2 geomagnetic model. Red line corresponds to the RcPR calculated when the dipole moment and local magnetic latitudes are considered in the Masarik and Beer[4] model. Dashed blue line provides the global average production rate when both local virtual dipole moments and magnetic latitudes are used (see Methods). The different small panels correspond to the increasing sum of harmonic contributions from the axial dipole to the harmonic degree 10. b) Global average RcPR based on the CALS10k.2 geomagnetic model using the dipole moment and the local magnetic latitudes for a sum of increasing harmonic contributions (see legend) from the axial dipole field up to the 10-degree full harmonic field. c) Relative differences of all the curves shown in b) respect to that given by the axial dipole field (values are given in %). Same color code for the harmonic terms of b). All curves have been normalized by the mean value.

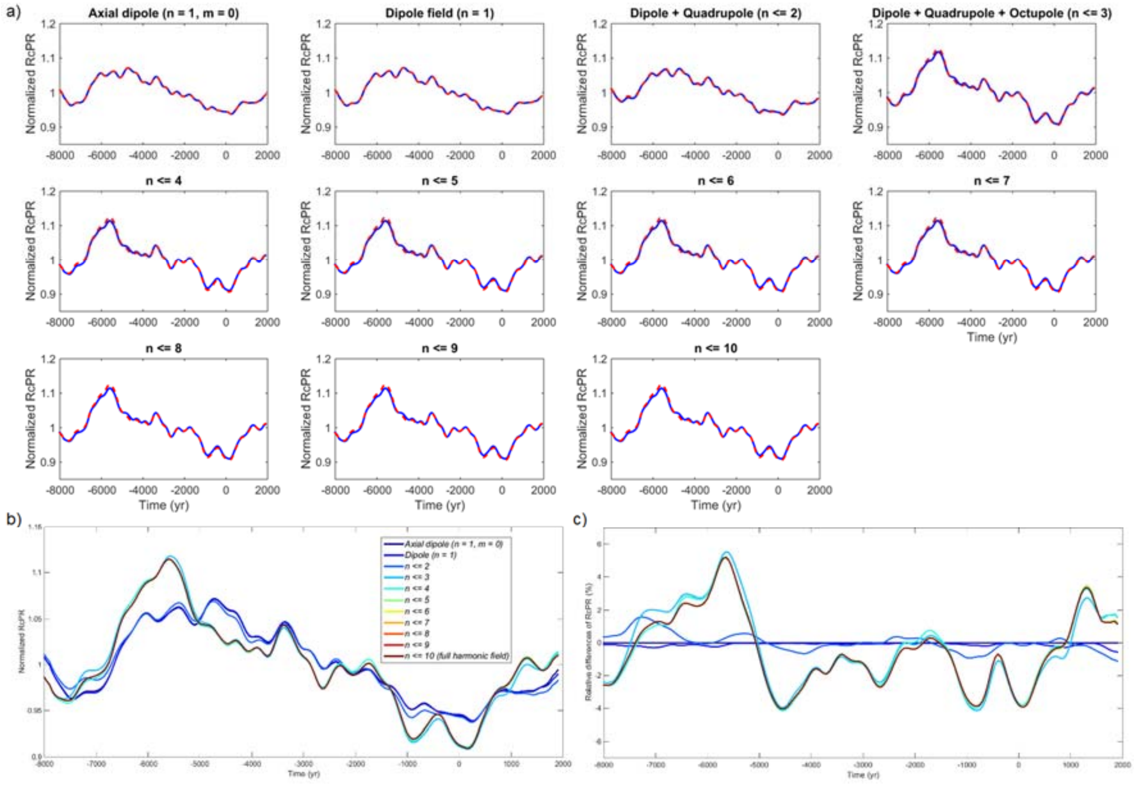

Figure 4S. a) Comparison of the production rates based on the SHA.DIF.14k model according to two alternative physical models: Masarik and Beer[4] in blue and Kovaltsov et al.[6] in red. b) Comparison of the intrinsic period histograms obtained from the radionuclide curve of Steinhilber et al.[23] and the radiocarbon curve of Roth and Joos[24]. The periods are obtained by bootstrap random iterations. Periods lower than 500 years are removed.

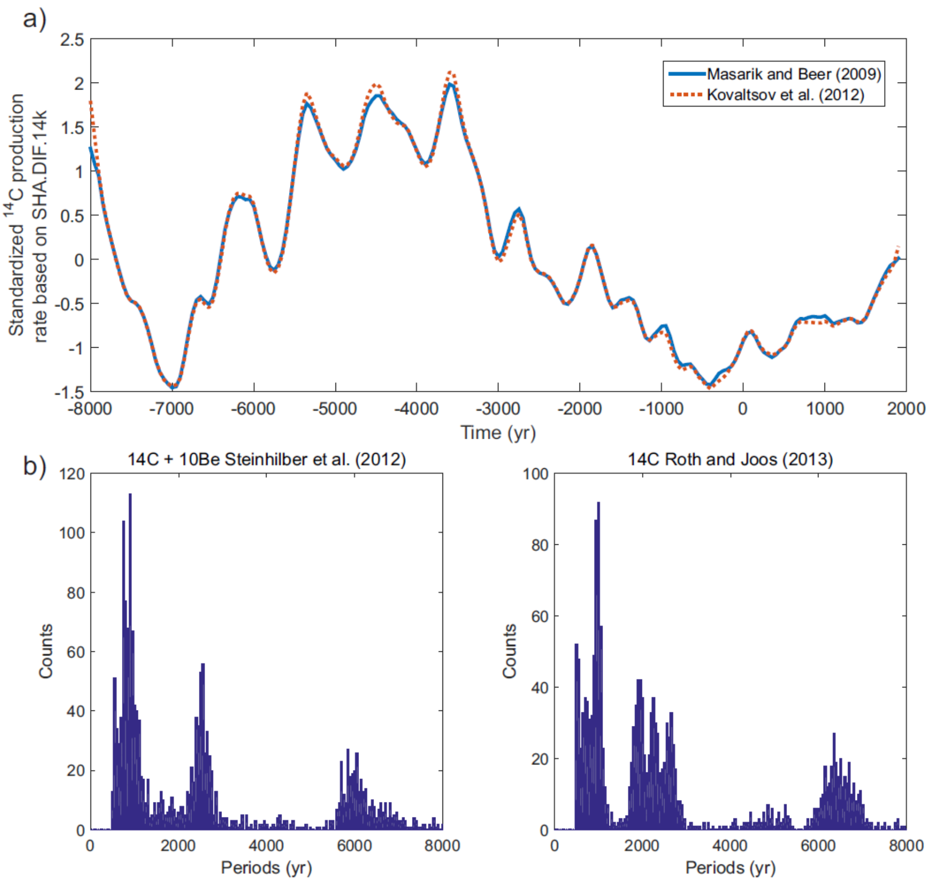

Figure 5S. Dependence of the RcPR with the normalized dipole moment (see legend) and the Earth's magnetic latitudes according to the physical model of Masarik and Beer[4]. Grey band shows the range of values of the dipole moment during the Holocene. Dipole moment values are normalized to the present value of $7.9 \cdot 10^{22}$ A · m^2.

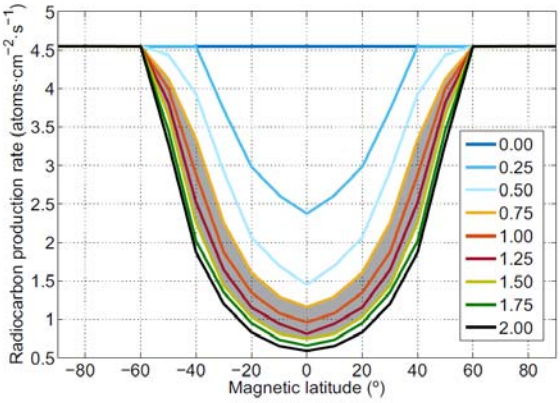

Supplement: Supplementary file 1 — Supplementary Material [file 41598_2018_28115_MOESM1_ESM.pdf]
